# Supplementary material for: Cloud BioLinux: pre-configured and on-demand bioinformatics computing for the genomics community
Source: BMC Bioinformatics. 2012 Mar 19;13:42. doi: 10.1186/1471-2105-13-42 (PMC3372431; doi:10.1186/1471-2105-13-42)
Supplement: Additional file 1 — Supplementary 1 Cloud BioLinux software documentation in the form of a mini, self-contained website. Users need to download and uncompress the .zip file, and open through a web browser the "index.html" file available on the main directory. (ZIP 1823 kb). [file 1471-2105-13-42-S1.ZIP › Cloud-BioLinux-Package-Documentation/docs/artemis.html]

Bio-Linux Software Documentation Pages

Back to search form

## artemis

|  |  |
| --- | --- |
| Name | artemis |
| Description | **Artemis** is a free genome viewer and annotation tool that allows visualization of sequence features and the results of analyses within the context of the sequence, and its six-frame translation.  **Artemis** is written in Java, and is available for UNIX, GNU/Linux, BSD, Macintosh and MS Windows systems. It can read complete EMBL and GENBANK database entries or sequences in FASTA or raw format. Extra sequence features can be in EMBL, GENBANK or GFF format.  The Artemis Manual can be found in PDF here as well online in HTML here |
| Homepage | http://www.sanger.ac.uk/Software/Artemis/ |
| Remote Documentation | http://pseudomonas-syringae.org/artemis\_tutorial.htm |

|  |
| --- |
| Please see Remote Documentation |
